# Antimicrobial Resistance: A Cause for Global Concern

**DOI:** 10.1186/1753-6561-7-S3-S1

**Published:** 2013-07-22

**Authors:** Rubina Lawrence, Ebenezer Jeyakumar

**Affiliations:** 1 Department of Microbiology and Fermentation Technology, Sam Higginbottom Institute of Agriculture, Technology and Sciences, Deemed-to-be-University, Allahabad, Uttar Pradesh 211007, India

## 

The three day conference on Antimicrobial Resistance: A Cause for Global Concern was organized from 6-8^th^ February, 2012 by the Department of Microbiology and Fermentation Technology, Sam Higginbottom Institute of Agriculture, Technology and Sciences (SHIATS) [Deemed-to-be-University], Allahabad, India. The conference received an overwhelming response from three hundred participants including scientists, medical professionals, academicians, industrialists and students from all quarters of the country. Participation from the Clinical and Laboratory Standards Institute, USA also made a remarkable contribution to the event.

Elaborating on the focal area of the event, Organizing Secretary and Convener Prof. (Dr.) Rubina Lawrence, spoke on the significance of miracle drugs in the treatment of infectious diseases. She emphasised the chemical repercussions of the chemical warfare waged against microbes through therapeutic and non-therapeutic uses of antimicrobial agents. She highlighted the global problem of antimicrobial resistance (AR) which is particularly pressing in developing countries where the infectious disease burden is high, there are cost constraints on testing to identify resistant infections and using newer and more expensive agents to treat them. In her address she illustrated the recent survey which emphasized that “Antibiotic resistance is a burgeoning problem in India”. Due to availability of over-the-counter antibiotics and their improper and rampant use, antibiotic resistance in the Indian population has skyrocketed. The future is however not entirely gloomy. The global strategy of WHO for the containment of antimicrobial resistance was underlined in her talk. However significant gaps in knowledge, research and development (R & D), funding and policy remain, particularly in developing countries. Prof. Lawrence, stated that the Second National Conference on Antimicrobial Resistance: A Cause for Global Concern is a follow-up to the first national conference organized in the year 2009 that was organized to further re-assess, consolidate the latest picture of the issue and the challenges faced thereof through the years. Concluding her talk she said that the conference is ventured to address this peril and to formulate strategies to combat it.

The popular consensus was that the conference succeeded effectively in attaining the various interlinked fields in contributing to analyse the problem of antimicrobial resistance. Presentations made under the five major subthemes are described below.

## Clinical challenges in addressing resistance to antimicrobial drugs in the 21^st^ century

**Dr. K. Venkatesan**, Scientist F/Deputy Director, National JALMA Institute of Leprosy and Other Mycobacterial diseases, Agra focused his talk on tuberculosis by stating it as one of the leading causes of mortality in India, killing two people every three minutes *i.e*., nearly 1,000 per day as reported by the Directorate of Health Services, India in 2009 while an estimated 2 billion people are infected with the pathogen worldwide. He quoted a study conducted on a cohort of patients from urban population in Mumbai reporting a high incidence of MDR-MTB (51%) [1]. He further expressed that a rise in MDR-TB cases, accounting to about 5% of 9 million of all types of TB in several countries, is a cause of great concern that threatens the success of DOTS (directly observed treatment, short course); the WHO recommended treatment approach for detection and cure of tuberculosis [2].

As the burden of MDR/XDR-TB is growing, strategies to combat its emergence include having clear policies, radical change in policies, optimal management of existing cases, steps to intensify R & D for rapid diagnostics and discovery of new drugs. He further stressed on the core policy packages of World Health Day 2011 in the commitment of a comprehensive, financed national plan with accountability and civil society engagement. This will help in strengthening surveillance and laboratory capacity, ensuring regular supply of good quality medicines, regulating and promoting rational use of medicines including animal medicines, ensuring proper patient and animal care, enhancing infection prevention and control in human and animals, fostering innovations, R & D of new technologies and medicinal products.

**Dr. Rama Chaudhary**, Professor, Department of Microbiology, All India Institute of Medical Sciences, New Delhi discussed epidemiology and risk factors for *Clostridium difficile* in cases of antibiotic associated diarrhoea (AAD) being the primary causative agent. She highlighted the significance of molecular characterization of *C. difficile* and stressed the need to screen for the organism in patients with antibiotic associated diarrhoea. Dr. Chaudhary concluded that analysis of *C. difficile* isolates from different geographical regions within the country should be done using a molecular approach to rule out the presence of the hyper virulent strain in the Indian setting.

The clinical and microbiological perspectives of ESBL infections were illustrated in the presentation of **Prof. (Dr.) K. N. Prasad**, Department of Microbiology, Sanjay Gandhi Postgraduate Institute, Lucknow. He stated that in India, bacteria are now producing multiple types of beta-lactamases. With limited treatment options available, there is a need to optimize the use of antibiotics against rapid proliferation of the bugs, since failure to do so will eventually lead to a pre-antibiotic era. The right drug, dose and duration were the 3 Ds explained by him for optimization. The antibiotic therapy was classified on the basis of a time dependent and concentration dependent treatment approach. He further stated that with the increase in minimum inhibitory concentration (MIC) in recent years, blood levels of antibiotics are not sufficient to remain above MIC for >40-60% time of dosing interval. Combinations of beta-lactamase inhibitors (BLI) along with cefepime and cefpirome deserve evaluation as alternatives to carbapenems in severe ESBL infections.

Prof. Prasad said that ESBL testing should be routinely done for epidemiological studies. Sensitivity testing for more than one third-generation cephalosporin should be done on a routine basis. He suggested that clinical microbiologists should encourage de-escalation and provide actual MICs for MDR pathogens. Discs available for all carbapenems should be tested since S/R of one carbapenem cannot extrapolate to another. Further, training should be given to clinicians on MIC breakpoints and optimization of dosage. Some of the steps to prevent infections elucidated by Prof. Prasad were barrier precautions during catheter insertion and care, surveillance for catheter-related infections and timely drain out of catheter line, to target the pathogen not the contaminant/colonizer, to practice empiric therapy to likely pathogens and target definite therapy to known pathogens. Some of the other measures highlighted by him were to consult infectious disease specialists/clinical microbiologists for patients with serious infections, stop unnecessary use of antibiotics, consider de-escalation to prevent co-lateral damage and to break the chain of transmission through hand wash or hand rub. He concluded his talk with an emphasis on awareness programs for patients to avoid use of expensive antibiotics and to promote hygienic and hand sanitation practices.

**Prof. (Dr.) Pallab Ray** from the Department of Medical Microbiology, Postgraduate Institute of Medical Research and Education, Chandigarh spoke on the pharmacokinetic and pharmacodynamic approaches to combating drug resistance.

To optimize treatment in critically ill patients with infections, Dr. Ray said that *in vitro* susceptibility of the pathogen holds equal importance to the right choice of antibiotic to solve the antimicrobial therapy puzzle. Taking into account the pathophysiological and immunological status of the patient, the selection of the right antimicrobial, timely administration, its route, dosage, schedule and duration is mandatory. He suggested that in most cases 7-10 days therapy is adequate and a shorter course with better clinical outcome can result in fewer subsequent superinfections with MDR pathogens. He further emphasized on the importance of procalcitonin as a biomarker to monitor the efficacy of treatment. He concluded his talk by stating that appropriate antimicrobials alone are not adequate for therapy; appropriate regimen should also be followed to reduce the emergence of drug resistance.

**Dr. Sanyog Jain**, Department of Pharmaceutics, National Institute of Pharmaceutical Education and Research, SAS Nagar, Punjab, drew attention to the nanodrug delivery system as a rational approach to combat multi-drug resistance in microorganisms. The current scenario of antimicrobial therapy demands a high degree of counter mechanism to prevent wide spread multiple drug resistance. One among the race can be the formulated strategy in which nanodrug delivery system can be efficiently exploited to combat multiple drug resistance, as suggested by Dr. Jain. He further stated that the novel formulations are capable of bypassing the most common route of multiple drug resistance and altered pharmacokinetics thus can achieve higher therapeutic efficacy at lower doses. Various nano-formulations such as liposomes or nanoparticles have been evaluated to establish the superior therapeutic outcome as compared to conventional therapy. Furthermore, the targeting potential of these new drug delivery systems can be selectively implemented to combat multiple drug resistance.

The lecture of **Dr. Sukla Biswas**, Emeritus Scientist, National Malarial Research Institute, New Delhi focused on the molecular mechanisms of antimalarial drug resistance. Higher malaria morbidity and mortality, increased cost of malaria case management, increased burden on the health care facilities, predisposition of malaria epidemics and increased relative prevalence of *Plasmodium falciparum* were outlined to be responsible for drug resistant malaria in India. The important aspects for the successful implementation of antimalarial drugs illustrated by her were: good tolerability, affordability, availability in endemic areas and short course regimens. As per the reports of Dr. Biswas, drug resistance is seen predominantly in *P. falciparum* as compared to *P. vivax*. The SP (sulphadoxine/pyrimethamine) failure rate in *P. falciparum* has increased from 7.7% during 1994-96 to 25.9% during 1997-2007. Her studies depicted that parasites isolated from the Nalbari and Dalu districts of Assam were highly polymorphic, though Nalbari isolates revealed relatively high diversity. A high degree of multi-clone infections could be an adaptive feature for parasitic survival in high endemic regions. High prevalence of drug resistance revealed mutations in *dhfr*, *dhps* and *Pfcrt* genes. For treatment of *P. falciparum* malaria, the ACT (artesimate + sulfadoxine – pyrimethamine) resulted in rapid parasite clearance well within a day or two with a cumulative cure rate of 95.3%. This drug regimen was well tolerated and concluded to be safe and effective. The prevalence of quintuple mutation (DHFR triple mutation and DHPS double mutation) can be used as a tool to screen clinical isolates by PCR based assay for monitoring SP clearance. Dr. Biswas explained that the emergence and spread of resistance is influenced by interactions between the malarial parasites, vectors, antimalarial drugs and humans. She further suggested that continued surveillance to analyse mutations of target molecules may also offer a useful tool in epidemiological surveys for drug resistance. She said that formulation of malarial treatment policies, measures to curb malaria morbidity and mortality and planning of cost-effective malaria control are some of the key areas to be focused on.

**Prof. (Dr.) Gopal Nath**, Head, Department of Microbiology, Banaras Hindu University, Varanasi said that the increasing trend of antibiotic resistance among superbugs has led to the search for alternatives like antimicrobial peptides, bacteriocins, probiotics and bacteriophages. Elaborating his talk on bacteriophage therapy, Dr. Nath divided the history of phage therapy into four periods: early enthusiasm, critical scepticism, abandonment and recent interest and reappraisal.

Although phage therapy trials in United States and most of Western Europe ceased after World War-II, probably due to widespread success and the availability of antibiotics, it is still being actively pursued in Soviet Union and some other Eastern European countries. Phage therapy has been tried extensively which is reflected by the fact that nearly 800 papers have been published during 1917-1957. Nevertheless, the reports were variable, which was explained to be due to the paucity of understanding of heterogeneity and ecology of both the phages and bacteria involved, failure to select phage of high virulence against the target bacteria before using them on patients and use of single phage in infections which involved mixtures of different bacteria.

Dr. Nath illustrated with the classical example of super bug *Pseudomonas aeruginosa*, which is notorious as it gains resistance from its environment. To achieve an effective control of infections caused by *Pseudomonas* sp., Dr. Nath isolated bacteriophages from different sources and checked their activity on different clinical isolates of *Pseudomonas aeruginosa*. On the basis of the dendogram constructed, phages with similar activity were identified and among them the most active viruses were selected to make a cocktail for the therapy of the mouse burn *Pseudomonas aeruginosa* septicaemia model.

**Prof. (Dr.) Varsha Gupta**, Department of Microbiology, Government Medical College Hospital, Chandigarh spoke on the epidemiological and therapeutic challenges of ESBLs in India. She explained that the pattern of ESBL infections differ in community and hospital with respect to the organism, type of ESBL, type of infection, molecular epidemiology and geographical breadth/epidemiology. The global figures projected by her showed that almost every part of the globe is affected by ESBL positivity. In the Indian scenario, ESBL was first reported in Nagpur during 1997, followed by several reports from Delhi and until 2009 Sanjay Gandhi Postgraduate Institute of Medical Sciences, Lucknow reported rates of up to 70% in *Escherichia coli*. Genotype studies of the ESBLs showed higher incidence of CTX-M followed by TEM with least incidence of SHV type of ESBL. However, reports on the global incidence of ESBL genotypes represent SHV, TEM, CTX-M and others (VEB, VER, BEL-1, BES-1, SFO-1, TLA and IBC). Dr. Gupta quoted a few genotypic studies that have been conducted in India (hospitals and community) during her presentation. In Southern India, SHV-12 was reported in *Klebsiella pneumoniae*. SHV-5 and TEM-104 in *Salmonella seftenberg* and *K. pneumoniae* isolates in Delhi and CTX-M-15 in *Escherichia coli* and *K. pneumoniae* in New Delhi and Coimbatore, respectively have been reported.

With the present drug resistance pattern, Prof. Gupta suggested that carbapenems are the first line agents which could be effectively used in the treatment. She said that no randomized control trials (RCTs) are available from India but data from case series and retrospective studies show positive results. These carbapenems also have a high inoculum effect and the members suggested were ertapenem, imipenem, meropenem and doripenem. Further beta-lactam and beta-lactam inhibitor combinations suggested by her were clavulanic acid, sulbactum and tazobactum. Tazobactum was found to be the most potent followed by clavulanic acid as they were active against TEM, SHV and CTX-M. The other drugs suggested by her were piperacillin and tazobactum with favourable results. Clavulante was shown to be a good inhibitor for detection of ESBL. She further said that though antibiotic combinations *viz*., amoxycillin - clavulanic acid and ticarcillin – clavulanic acid, showed borderline activity, they did not work when ESBL and *AmpC* together were found in the organism. Clavalunate was found to be effective against CTX-M-15 and not against OXA-1 even though CTX-M-15 and OXA-1 are determined by the same plasmid *i.e.* common occurrence. Tigecycline has been approved by US FDA for complicated intra-abdominal infections (IAIs) and skin and soft tissue infections (SSTIs). Limited clinical data is available from India where tigecycline is not used for UTI since it is not excreted in the urinary tract. Moreover, after intravenous infusion, rapid tissue distribution occurs; hence it is of great concern when used in blood stream infections. In ventilator associated pneumoniae (VAP), imipenem is better than tigecycline. Her lecture depicted that despite *in vitro* susceptibility of cephalosporins, worst outcome was found and hence not recommend for ESBL infections. She emphasized that aminoglycosides, fluoroquinolones and cotrimoxazole should be used with caution in serious infections even after documentation of *in vitro* activity. Fluoroquinolones are an excellent option for UTI patients with ESBLs if the organism is susceptible. However, aminoglycosides should never be used in monotherapy but can be useful as adjuncts. There is no CLSI breakpoint for susceptibility of colistin against *Enterobacteriaceae* but E-test has been proposed. She said that some of the newer drugs like fosfomycin, temocillin and pivmecillinam are unfortunately not available in India. Fosfomycin is approved by US FDA for treatment of uncomplicated UTI. *In vitro* studies show that fosfomycin is active against ESBL producing *E. coli* and *Klebsiella* sp. Fosfomycin E-strip has been used by Dr. Gupta against ESBL for laboratory testing. Temocillin is a derivative of ticarcillin and is stable to beta-lactamase action, well tolerated, and licensed in the UK and Belgium. Similarly mecillinam is also under testing in her laboratory. She stated that other carbapenems like biapenem and feropenem are soon to be available in the market.

Dr. Gupta further suggested that control of infections from the reservoirs like GIT, orpharynx, colonized wounds, urine and community with ESBLs should be done. Measures should be taken to adopt proper infection control practices such as: hand washing, barrier precautions, isolation of colonized or infected patients, surveillance of patients of ICUs, early detection, antibiotic restriction and antibiotic restriction strategies. She concluded by stating that ESBLs are distributed world-wide and the major challenge is therapy of severe infections.

The lecture of **Dr. Deepa Bisht**, Scientist C, Senior Research Officer, Department of Biochemistry, National JALMA Institute of Leprosy and Other Mycobacterial Diseases, Agra was focused on the proteomic approach for identifying drug targets in mycobacteria. She explained that using a proteomic approach, identification of drug targets, development of vaccines and diagnostics for tuberculosis can be achieved. Dr. Bisht illustrated that as per the estimates of WHO, [2], 9.27 million new cases were reported world-wide with 1.8 million deaths. India accounts for one fifth of the global incidence with 1.9 million new cases and 0.3 million deaths. The enormity of the problem has been further worsened by widespread development of MDR-TB and XDR-TB. She stated that the prevalence of MDR-TB is about 3% in new cases and 12-17% in re-treatment cases in India. Treatment success rates have tripled from 25-86% and death rates have been cut 7-fold from 29% to 4% in comparison to the pre-RNTCP (Revised National Tuberculosis Control program) era.

She said that aminoglycosides are major second line anti-tuberculosis drugs for treatment of resistant tuberculosis. In order to understand the biology of *M. tuberculosis*, proteomics could be used as a complementary tool for genomic comparisons. The challenges for proteomics outlined by her were analysis of low abundance proteins and protein interaction studies, where native conformations of proteins must be maintained to obtain meaningful results. She stated that most of the proteomic approach relies on methods that are not high-throughput methods. New technologies will have to emerge before protein analysis on a large scale becomes a reality.

## Antibiotics, agriculture and super bugs

**Prof. (Dr.) Brahma Dutta Kaushik**, Dean, College of Biotechnology, Anand Engineering College, Agra addressed his keynote lecture on the topic “Next generation super bugs; Microalgae as key unexplored reservoirs of bioactive metabolites”. Prof. Kaushik in his lecture described the significance of cyanobacteria by stating that it produces a number of secondary metabolites, allelochemicals and enzymes which exhibit bioactivities such as inhibitory properties against microorganisms and toxicity to invertebrates and vertebrates. These metabolites can be used for development and application as algicides, fungicides, herbicides and insecticides. Such allelopathic compounds have various modes of action: from inhibition of photosynthesis to oxidative stresses or cellular paralysis. However, presently the use of these metabolites in agriculture is limited. He said that the cyanobacterial metabolites (cyanotoxins) are allelochemicals which provide a competitive advantage to the producer, are novel compounds with diverse bioactivities, act as an antifungal agent (*i.e*., with bio-control properties) and biopesticides and also possess cytotoxic properties (*i.e*., with anticancer or antitumor agents).

While concluding his lecture Dr. Kaushik said that Cyanobacteria are key to any understanding of the Earth's early biology and environmental history. He stated that developing tight linkages with crop plants is needed for efficient nutritional interactions, thus improving plant productivity. Gene mining can help to explore and excavate novel genes and bioactive molecules. Cyanobacteria are natural candidates for seedling inoculation, especially in reforestation and rehabilitation of coastal systems, and need to be exploited.

The second key note address was presented by **Dr. Debashish Chattopadhyay**, Additional Director (Microbiology), National Centre for Disease Control, New Delhi. He spoke on “Antibiotic resistance markers in genetically modified plants: An evaluation of possible human risk”. The sunny sides of genetically modified foods explained by him were: plants with increased yields, nutrient content, herbicide tolerant plants, insect resistant plants, animals with a better yield of products (*e.g*. milk, meat), plants producing drugs, vaccines and lower levels of greenhouse gas emission. On the dark side he emphasized the safety issues like deletion/suppression of useful nutrients, insertion/activation of anti-nutrients, insertion/activation of allergens and the potential transfer of the antibiotic resistant marker (ARM) gene in GM food to the human body through incorporation into gut flora.

The risk groupings of ARMs as discussed by him were Group I, Group II and Group III. Group I (*e.g. npt II*) is of very little clinical utility and has high prevalence in the gut. Neomycin phosphotransferase II (*npt II*) confers resistance to the aminoglycoside group of antibiotics *e.g.* neomycin (used for gut sterilization) and kanamycin (little clinical application); both of which have high prevalence of resistance in the gut and environment. In Group II the significance of *bla* (beta lactamase) and *aad-A* (aminoglycoside-o-nucleotidyl transferase) were discussed. He explained that *bla* mainly confers resistance to ampicillin and penicillin, has moderate clinical application and moderate prevalence in the gut and environment [3]. The *bla* gene product β-lactamase hydrolyses penicillin. He further added that there is a chance of the emergence of ESBL production by bacteria under selection pressure of continued use of this category of antibiotics, therefore there is a requirement for a newer generation of antibiotics *e.g.*, carbapenems. On the other hand, *aad* confers resistance to streptomycin which is still the drug of choice for tuberculosis in the resource-poor developing countries and spectinomycin which is the second line treatment for anogenital and joint infections due to *Gonococcus. npt III* is included under Group III. This has higher clinical utility and low prevalence in the gut and environment. This marker is used for experiments only and not for production of GM crops. He mentioned that the overall risk of transfer of antibiotic resistance through DNA is minimal due to the negligible quantity and heterogeneity of source DNA, extremely low rate of its transfer under experimental conditions, loss of integrity during food processing, passage through the GI tract and so on.

**Dr. Gerard Abraham**, Principal Scientist, Centre for Conservation and Utilization of Blue Green Algae, Indian Agricultural Research Institute, New Delhi presented his lecture on antimicrobial activity of *Azolla microphylla*. He said that seasons play an important role in the secondary metabolite profile of *A. microphylla*. In his study, extracts of *Azolla microphylla* showed significant antibacterial but no antifungal activity; suggesting that this organism can be exploited as a source for antagonistic compounds. However, the proteomic profile needs to be investigated to further understand the involvement of other molecules in antagonistic response.

**Dr. Bhoj Raj Singh**, Principal Scientist, Joint Directors Lab, Centre for Animal Disease and Diagnosis, Indian Veterinary Research Institute, Izatnagar, Bareilly presented a thought provoking issue *i.e*., “The positive aspect on the emergence of antibiotic drug resistance (ADR) in the existence of life on earth”. In the lecture, Dr. Singh suggested that bacteria are the major scavengers that play a significant role in keeping the earth liveable; their survival is probably more important than ours, that is why nature has protected them through the development of ADR.

**Dr. Rajesh Kumar Vaid**, Senior Scientist (Veterinary Public Health), Veterinary Type Culture Centre, National Research Centre on Equines, Hisar spoke on antimicrobials and their judicious use in livestock. He said that antimicrobial use in food animals can lead to selection of antimicrobial resistant zoonotic enteric pathogens. These are transferred to people *via* food or animal contact. Further, resistant bacteria excreted in the faeces of animals having received antimicrobials serve as a reservoir in the environment. These are the issues in antimicrobial resistance in veterinary medicine as outlined by Dr. Vaid. The factors that may contribute to the antimicrobial resistance as elaborated by him were indiscriminate use of antimicrobials in animal agriculture/aquaculture which may contribute to the environmental reservoir of resistant microbes, trend towards intensive livestock production, more animals per farm and susceptible animals in close physical contact. In addition, increasing international travel and trade can also add to this problem. However, there are controversies with regard to concerns on the impact of use of antimicrobials in animals on public health. Dr. Vaid stated that antimicrobials used in livestock to control *Salmonella* and *E. coli* are of great concern as antibiotic resistance developed in these pathogens are subsequently transferred to humans through contaminated food. He concluded his lecture by saying that stringent adoption and enforcement of OIE (Office International des Epizooties) guidelines/List of AMs in Veterinary medicine/CE credits should be done, training and application of international/standard laboratory procedures in antimicrobial susceptibility testing should be adopted, there should be increased surveillance, monitoring and reporting of anti-microbial drug resistance-epidemiological cut-off values from microbes of veterinary origin. He also recommended to focus research particularly on organisms like *Salmonella*, *Escherichia coli*, *Klebsiella*, *Staphylococcus*, *Streptococcus*, *Pasteurella* spp. *etc.* He added that research on discovery and application of biosecurity, hygiene, vaccination, probiotics, prebiotics and essential oils for growth promotion should be encouraged. Research on novel antimicrobials such as RAMPs (receptor activity-modifying proteins) like defensins should be conducted.

The presentation of **Dr. Ravinder Kumar**, Department of Biotechnology, KVA DAV College for Women, Karnal was a depiction on the distribution of multiple antibiotic resistance genes and pathogenic factors of *Staphylococcus aureus* isolates that has raised the alarming situation on the safety aspects of multiple antibiotic resistant pathogens. He concluded that the projected genetic frame of *S. aureus* isolates could be used for developing further evaluation strategies and control measures.

**Dr. Satyendra Gautam**, Head, Food Science and Safety Section, Food Technology Division, Bhaha Atomic Research Centre, Mumbai elucidated the idea of targeting the pathways involved in drug resistance or by inducing genetically regulated programmed cell death (PCD) like process to control emerging antibiotic resistance in pathogenic microbes.

**Dr. Nivedita Sharma**, Department of Basic Sciences, Dr. Yashwant Singh Parmar University of Horticulture and Forestry Nauni, Solan, described the food bio-preservative potential of antimicrobial bacteriocins. She said that food safety is an important issue of international concern. Bio-preservation of food has emerged as an attractive and safe approach for consumers. She said that among biopreservatives, antimicrobial bacteriocin biomolecules have caught the attention of food scientists due to their unique properties of inhibiting food-borne pathogens and spoilage-causing microorganisms without expressing any resistance against them. In nature, many microorganisms are capable of secreting novel bacteriocins exhibiting strong antagonism. However this potential of microorganisms has largely been untapped and so far only nisin has got the commercial license for food preservation. Thus, there is an urgent need to explore the potential of different bacteriocin producing strains belonging to lactic acid bacteria as well as *Bacillus* spp. for the effective biopreservation of food. She further highlighted some of the bacteriocins produced in their research laboratory from different organisms. She reported the effectiveness of lenticin, brevicin and subtilin against pathogenic bacteria *viz.*, *Listeria monocytogenes*, *Staphylococcus aureus*, *Aeromonas hydrophila*, *Clostridium perfringens*, *Salmonella typhimurium*, *Leuconostoc mesenteroides*, *Enterococcus faecalis*, *Pseudomonas aeruginosa* and *Escherichia coli*. Further, the efficacy of lenticin and nisin as biopreservative in cheese inoculated with pathogenic bacteria was illustrated. With the food safety point of view, she recommended the use of bacteriocins as bio-preservatives.

## Emergence, spread and environmental effect of antimicrobial resistance

**Dr. Rishi Shanker**, Head, Environmental Microbiology Division, Indian Institute of Toxicological Research, Lucknow presented the keynote address on the topic “Pathogenic bacteria and resistance to antimicrobial agents: environmental sinks to aquatic landscapes”. He started his address by quoting the Indian scenario, that from the 23 macrocities, approximately 1500 million litres of sewage/day is released and only 10% of them are treated. As recorded by the Central Bureau of Health Intelligence, Ministry of Health and Family Welfare, there were 10.5 million cases of diarrhoea in 2003. Approximately less than 30 million lives are lost annually due to water related disease in India as per the estimates of the World Bank.

Dr. Shanker stated that the microbiological quality of water in the developing world in rural areas is poor due to contamination by surface water run-offs, sewage sludge, animal waste and lack of chlorine treatment. However in the urban areas, the poor quality water is due to inadequate filtration and chlorine treatment, proliferation of bacteria along the distribution system, inadequate sewage systems, recreational use of surface water, ground water contamination by septic effluent and no treatment of urban storm water. The water sources contain total coliforms comprising of faecal coliforms, *Escherichia coli* and pathogenic *Escherichia coli* (EHEC, ETEC, EPEC and EAEC). He explained that *E. coli* being an indicator bacteria gets transmitted through ingestion of contaminated water by human, meat, unpasteurized milk, water, fruits and vegetables. For diarrheagenic *E. coli*, EHEC, EPEC and ETEC the infectious dose is >10-200 cells.

He explained that for drug resistance and virulence gene profiling of water quality, indicators *E. coli* and *Enterococci* in surface waters are used. In a study conducted in their laboratory, the occurrence of different types of *E. coli* were isolated from water collected from different sampling sites of the rivers Gomti, Saryu and Ganga. They observed that pathogenic virulents of water quality indicator were present in potable as well as surface waters of the river Ganga and its tributaries Gomti and Saryu. Using PCR based fingerprinting in *E. coli* isolates from surface water of the rivers Gomti and Ganga, they could locate the position of virulent genes. Antimicrobial resistance systems in enterotoxigenic *E. coli* revealed multiple drug resistance in this most deadly strain of *E. coli* prevalent in low hygienic areas, slum areas and urban-rural interfaces. The markers like LT-1 and LT-2 in the genes were found to be most common in these three rivers and hence were used for further studies. Dr. Shanker said that the labile toxin (LT-1) profile is present in approximately 68% of the subjects. Without any seasonal variation LT-1 toxin producing ETEC are present all through the year. LT-1 gene is a target for culture independent quantitative detection by real time PCR probes. Further in their studies it was also reported that the aquatic flora from Gangetic riverines have been found to harbour high levels of ETEC. They act as secondary reservoirs and release this organism from time to time in water bodies. Similar incidence was also observed in vegetables grown on the river bank and sediment samples of the river Ganga detected by common probe system. Further profiling of *Enterococci* as an indicator organism in their studies revealed that MDR isolates could be recovered from different sites. From the PCR based profiling of *Enterococcus* sp. heterogeneity in the river Ganga with the order of *E. faecalis* followed by *E. faecium*, *E. durans*, *E. hirae* and other *Enterococci* was revealed.

**Dr. Anita Arora**, Microbiology consultant at the Fortis Escorts Heart Institute, New Delhi illustrated the high prevalence of Extended Spectrum Beta-lactamases (ESBL's) in Indian Medical Centres and multiple mode of resistance mechanisms. She explained that ESBL's are associated with high mortality due to inappropriate choice of antibiotics. This would eventually increase carbapenem use and may select resistance to these key drugs. She concluded her talk with a call for efficient infection control and implementation of other intervention strategies.

**Dr. V. S. Randhawa**, Professor at the Department of Microbiology, Lady Hardinge Medical College, New Delhi explained that the prevalence of antibiotic resistant strains is generally proportional to the extent of use of any particular antibiotic in the area. In the study undertaken by him, the trends of ciprofloxacin resistance in *Klebsiella pneumoniae* isolates from paediatric septicaemic cases were discussed. The possible reasons for increased usage of ciprofloxacin figured out by him were due to change of drug of choice for typhoid fever, treatment of fever cases suspected for typhoid without investigation, empiric use of ciprofloxacin without isolation of the organism and performing susceptibility testing and decrease in the cost of tablet. Dr. Randhawa recommended the surveillance of antibiotic use at the national level, sales of antibiotics from hospitals and drug stores to be available from institutions, cities, states, countries and continents, monitoring the prevalence of antibiotic resistant bacteria, rational and judicious use of antibiotics, and use of synergistic combinations especially in complicated situations. He further said that risk factors for ciprofloxacin resistance as per long term use (> 20 days), dose (250 mg/bds) and hospital infection control, should be considered to prevent spread of ciprofloxacin resistant strains.

**Prof. (Dr.) A. J. Tamhankar**, Founder, Indian Initiative for Management of Antibiotic Resistance, India spoke on the elimination of antibiotic by sorption, photolysis, hydrolysis and biodegradation. He explained that antibiotics belonging to the group aminoglycosides and β-lactamase are readily degradable while tetracyclines and quinolones undergo slow degradation when sunlight exposure is limited. However, macrolides and sulphonamides are least susceptible for degradation. He reported that the hospital effluent waste water contains antibiotics like ciprofloxacin, sulphamethoxazole, erythromycin, norfloxacin, gentamycin and levofloxacin in ng/ml to μg/ml concentrations. He concluded that one has to be cautious about the phenomenon of antibiotic resistance as it is an evolutionary phenomenon, besides; human activity is adding and generating resistant bacteria to the environment.

**Dr. Poornima Vajpayee**, Scientist at the Environmental Microbiology Division, Indian Institute of Toxicological Research, Lucknow delivered a talk on the environmental reservoirs of bacteria resistant to β-lactam antimicrobials. She said that since antibiotics enter the environment from farms, pharmaceutical factories, homes and hospitals, antimicrobial resistant bacteria in the environment are a matter of concern. DNA carrying antimicrobial resistant genes could be released to the environment after bacterial death and can persist longer in the environment. Aquatic landscapes in populous countries are reservoirs to antimicrobial resistant pathogenic microbes due to indiscriminate use of antimicrobials in human and veterinary medicine and addition of untreated sewage and other domestic wastes from nearby planned and unauthorized settlements. Dr. Vajpayee reported isolating *bla_TEM_* gene in surface water collected from different sites in the river Gomti, in street water, potable water and aquatic weeds suggesting that *bla_TEM_* resistant genes may emerge as major contaminants in Gangetic plains in India. She said that aquatic ecosystems as well as potable water serve as environmental pools of β-lactam resistant genes that can potentially be transferred to pathogens.

In her concluding remarks she stated that the information on environmental reservoirs of genes conferring resistance to antimicrobials of the β-lactam group prevalent in the Gangetic plains will be useful in formulating strategies to protect the public from the menace of clinical risks associated with antimicrobial resistant bacteria.

## Computational modeling and the pro-drug approach to combat antibiotic resistance

**Prof. (Dr.) Indira Ghosh**, Dean, School of Computational and Integrative Sciences, Jawaharlal Nehru University, New Delhi addressed on the *in silico* study using pathways to inhibit the pathogenic bacteria, *Mycobacterium tuberculosis*. She said that drug development is difficult, costly and time consuming; however, new antimicrobials are needed to treat infections caused by ESKAPE (*Enterococcus faecium*, *Staphylococcus aureus*, *Klebsiella pneumoniae*, *Acinetobacter baumannii*, *Pseudomonas aeruginosa*, *Enterobacter* spp.) pathogens that currently cause the majority of hospital infections. According to IDSA (Infectious Disease Society of America) the decreasing investment in antibacterial drug development coupled with the increase in antimicrobial resistance represents an “IMPENDING DISASTER”, she quoted. Further, the challenges of anti-tuberculosis drugs explained by her were: lengthy and complicated nature for treatment of tuberculosis (*i.e*., 6-9 months), a course of four drug combinations (2 months = isoniazid, rifampicin, pyrazinamide, ethambutol; 4-7 months = isoniazid and rifampicin) often leads to low adherence (MDR & X-DR TB), treatment of TB-HIV co-infection, drug–drug interaction between anti-TB and antiretroviral (ritonivir) to counteract the effect of rifampicin, treatment of persistent and latent TB infection, prevention of active infection in high risk populations (people in contact with TB infected or HIV positive people) and treatment of pediatric patients. The goals of current anti-TB development elucidated by her were to shorten and simplify treatment for drug sensitive and MDR/XDR-TB, TB co-infected with HIV that can be treated with anti-retrovirals and latent TB infection. Besides these, improvement of efficacy and safety of drugs, usability in multiple populations and affordability were also stressed upon. She stated that the drug discovery approaches in TB should include genetic approaches to target identification (microarray expression analysis: to profile cellular responses to perturbations or environmental stress, whole genome sequencing), high throughput screening (whole cell [MIC] or enzyme based [IC50] assay, HTS for known targets, structural biology and virtual screening (docking: TB structural genome), ligand-based approaches (QSAR [quantitative structure-activity relationship], HQSAR [hologram QSAR], 3D QSAR like CoMFA [comparative molecular field analysis] and pharmacophore built using machine learning techniques. She informed the activities of TAACF [Tuberculosis antimicrobial acquisition and coordinating facility] and TB alliance in this regard. She further explained the strategies to kill an organism using metabolic pathways comprising of two methods. Firstly, by chemical flux through an essential metabolic pathway, microbial load can be decreased to the point where life is no longer possible. Secondly, an increase in the metabolic concentration due to stop in enzyme activity to toxic levels for the bacterium without harming the human.

The alternative targeting strategies from the recent studies conducted in her laboratory as laid down by Prof. Ghosh included using a comprehensive inhibitor of ICL (isocitrate lyase) along with inhibition of ICD (isocitrate dehydrogenase) – kinase. She explained that inhibition of ICD-kinase would increase the amount of active ICDs thus directly improving the efficacy of competitive inhibition of ICLs by reducing the available isocitrate in branch pathways. ICD-kinase as a potential target for drugs against persistent *Mycobacterium* was also identified. Some of the *in silico* systems biological approaches that assist in identification of various novel drug targets in various pathogens outlined by her were: Flux Balance Analysis (FBA), Metabolic Control Analysis (MCA), Bayesian Analysis, Load Point and Choke Point Analysis, Comparative Genomics and Microarray Analysis. She finally concluded by stating that biology has traditionally followed a reductionist approach in which individual components of a living system are studied separately; we need to reverse the process and study how these components interact to form complex systems using an integrative approach.

By quoting “Bad bugs need new drugs” **Dr. Girish Mahajan**, Senior Group Head, Prokaryote Technology and Antiinfective Discovery Division, Department of Natural Products, Piramal Life Sciences, Goregaon, Mumbai continued the lecture, expressing the antibiotic market to be very lucrative. He said there is an increasing demand due to global spread of the MDR pathogen, emergence of new virulent strains, the growing number of elderly and immuno-compromised patients and the difficulty to treat infections. He emphasized on microbial genomics as a guide to drug discovery and structure elucidation as it is a complementary tool to spectroscopy and enables rapid determination of complex structures. He further illustrated the genome analysis of compounds produced by *Streptomyces aizunensis* NRRL B-11277 from literature. This genome analysis has helped in identification of a novel antifungal compound [ECO-02301]. The same compound has been isolated in their laboratory from one of the isolates. The MIC of ECO-2301 was not at par with amphotericin-B. Further, there was evidence of cytotoxicity which hindered its further consideration for development. He also said that genomic approaches may take longer time as a way to identify the novelty in the final bioactive compound.

The second approach discussed by him on antimicrobial drug discovery was mutation. For this he showed an example of *Amycolata autrophica*. The organism was initially rejected because of lack of antimicrobial activity. However, treatment of inactive wild strains then discarded in the screening program was subject to intercalating agents ethidium bromide or daunorubicin. This induced the culture with very high frequency progeny which exhibited antibacterial activity. Antibacterial antibiotics 167-A and 167-B were isolated from the fermentation broth of mutant EB5 derived from an inactive wild strain of *Amycolata autrophica* 167 by treatment with EB. The antibiotic 167 B was identified as cervinomycin A2, component 167-A being a new representative of the cervinomycin group. A large number of mutants had to be screened with the mutation process, which according to Dr. Mahajan was the limitation of the method. The next approach found to be more fruitful as addressed by Dr. Mahajan was isolation of novel compounds from microbes in the marine environment. *Salinispora* sp., a Gram positive aerobic non-acid fast bacteria isolated from marine sediments, was reported with potential of compounds with anticancer, anti-inflammatory, antibiotic and antiviral activities. He exemplified the same by reporting the discovery of potent novel antibiotics (PM181104 and PM181108) from marine resources by his organization. Dr. Mahajan concluded his talk by saying that India is a rich source of diverse microbes owing to its large variations in climatic conditions and geographical extremes. This asset has to be used especially by collaborative research work with universities and industrial research organizations towards getting novel compounds. The active synthetic derivatives or microbial compounds should be tested on panels of hundreds of test cultures to strengthen their true trait; a strategy which may put us in the frontiers of global drug research.

The term homeoinformatics was introduced by **Mr. Suchir Arora**, Senior Consultant, Business and Decision, Gurgaon in order to model *Homo sapiens* biological systems against antimicrobial resistance through homeopathy. He said that drug interactions of allopathic drugs are well known, however for homeopathic drugs it has to be developed since the molecules are not characterized. He explained that conventional medications have options for personalized medicines through the help of pharmacogenomics. But in the case of the homeopathic approach the genetic, physical, emotional and personal health history *etc.* are followed. Hence more emphasis on individualized medicine is given. For the homeopathic approach, there is evidence from *in vitro* and *in vivo* streams facilitating our knowledge but much less work is done at *in silico* level. So effort will be made to establish a new stream, “homeoinformatics”, which will be a combination of bioinformatics and computer aided modelling. Application of bioinformatics such as effective data mining, network biology, systems biology *etc.* will be effective in understanding the principles of homeopathy for more effective treatment. He concluded by stating that in the primary steps computational modelling of homeopathic drugs is required to combat disease with a special focus on antimicrobial resistance. Later, we can exploit the possibility of establishing new principles, algorithms, strategies in providing evidence based support to the principles of homeopathy.

## Regulatory issues of antimicrobial resistance

The Co-Chair of the CLSI Sub-Committee on Veterinary Antibiotic Susceptibility Testing and Microbiology Technical Advisor at Elanco Animal Health, UK **Dr. Shabir Simjee** delivered the first keynote lecture of this session on harmonization in the generation, presentation and application of antimicrobial susceptibility test data for bacteria of animal origin: the X08 report. He highlighted on risk assessment, risk management and government legislation. He explained that the data generated from the surveillance programs are used for registration purposes, as an indicator for the emergence of resistance and, for national risk assessment and subsequent risk management guidelines. Dr. Simjee said that it is important to ensure that the data being generated is of uniform quality and is interpreted using single interpretive criteria. Pharmaceutical products should have risk assessment prior to registration or post-registration. He concluded that India does not have a risk assessment process for drug companies to submit presently but that could change. It is not possible to compare resistance rates from different surveillance schemes (horizontal comparisons) as they are not measuring the same parameter. Within national surveillance schemes (vertical comparisons) methods of analysis have changed overtime such that percentage resistance values need to be viewed with caution. Further he demonstrated that bacterial resistance showed varied antimicrobial susceptibility with respect to the source from where they are isolated *e.g*., *Escherichia coli* of humans will show a different antimicrobial susceptibility profile as compared to birds and so on.

Resistance interpretation is done on the basis of the clinical break point and epidemiological cut-off value (ECV). Because of discrepancies in interpretation of data, the CLSI initiative on harmonization was undertaken. CLSI publishes three types of documents *viz.*, standard, guidelines and report. For interpretation using the standard method the details are to be strictly followed, with the guidelines some modifications can be made in methodology, while the report is a technical document that is intended for informational use only and which offers guidelines on how it should be done. He emphasized that the need for development of X08 – R is driven by the needs of the VAST (Veterinary Antimicrobial Susceptibility Testing) group, confusion in use of common terms such as susceptible and resistance and use of wild type or epidemiological cut-off values for reporting results of national surveillance program. The benefits of using ECV as outlined by him were: early detection of resistance development in a given microbial population, it is easier to develop than clinical breakpoint, can be developed for any agent regardless of clinical usage and can be incorporated into all clinical breakpoints. However it may not be predictive of clinical outcomes and methods for setting ECVs are not yet standardized.

Dr. Simjee said that the clinicians look for clinical breakpoint without caring for the wild and non-wild type population. However, when the data on surveillance is observed over a period of time clinical breakpoint is of no value. Further to observe the trends, long term surveillance programs are needed. So he recommended from the X08 report the use of ECV rather than clinical break point value. He further explained the need to have universal adaptation of terminologies, and differentiation of ECV and clinical break points. Further, data obtained from Europe and the United States should not to be taken at face value, as it can be challenged, because the resistance and susceptibility standards of these countries vary.

**Dr. K. K. Tripathi**, Advisor, and Member, Review Committee on Genetic Manipulation, Department of Biotechnology, Govt. of India, New Delhi presented the second keynote address of this session. He spoke on the regulation of genetically modified plants and use of antibiotic resistance markers. He said that the various genetically modified organisms and r-DNA products are governed by the Environmental Protection Act 1986, Industries Development and Regulation Act 1951, Drugs and Cosmetics Act 1940 and Pharmaceutical Policy 2002. He also talked about the various biosafety frameworks in India which control the rules for manufacture, use, import, export and storage of hazardous microorganisms/genetically engineered organisms or cells (1989) under the EPA 1986 [4]. These rules are very broad in scope and cover the entire spectrum of activities relating to research, development and use of Genetically Modified Organisms (GMOs) and their products. He said that the regulatory system in India contains well defined guidelines and documentation to generate quantitative biological, ecological and agronomic supportive data. There are multidisciplinary experts as reviewers at various stages of the development process. An extensive safety assessment process is conducted in compliance with international agreements and harmonization with Codex/FAO/WHO. It also aims to ensure that GM crops pose no risk to human and animal health, environmental safety and agriculture productivity. He said that the antibiotic resistance markers are used in genetic engineering as a tool for recognizing the successful introduction of a new gene into a cell. Most antibiotic resistance marker genes confer resistance by inactivating the antibiotic through modifying enzymes. These antibiotic resistance genes are ubiquitous in the environment which is isolated from existing bacteria prevalent in the environment. Some of the benefits of using antibiotic resistance markers for GE crops as counted by him were: ideal selection system, simple selective procedure, does not affect the physiology of cell, is cost effective and readily available. However, the biosafety concerns of these include risk of horizontal gene transfer from GM plants to micro-organisms particularly genes conferring resistance to antibiotic. Though, horizontal gene transfer (HGT) across different bacterial taxa is well known, no examples of transfer of an antibiotic resistance determinant from transgenic plants to bacteria have been found.

To date there is no well-defined mechanism for the transfer of genes from plant cells to bacterial cells [5]. He concluded his lecture by stating that as per the safety assessment of antibiotic resistance in GM crops the decision of the International Food Biotechnology Council, Food and Drug Administration (FDA), Environmental Protection Agency (EPA), World Health Organization (WHO) [6], The European Commission Scientific Committees for Food and Animal Nutrition, The Nordic Working Group on Food Toxicology and Risk Assessment and European Food Safety Authority (EFSA) states that the likelihood of transfer of antibiotic resistance genes from GM plant to microorganisms, gastrointestinal tract microbes of man or animal, and in the environment is remote.

**Dr. G. J. Samathanam**, Advisor and Head, Department of Development and Transfer Division, Department of Science and Technology, Govt. of India, New Delhi spoke on the regulatory issues related to drug development and emergence of multi-drug resistance in modern antibiotics in the Indian context. He explained the key aspects of right to health and compared it with the data with respect to the economy, shifting pattern of global pharmaceutical market, products, therapy forecast and decadal progress of the pharmaceutical industry. He also presented an overview of the growth of the Indian drug industry and predicted the possible growth by 2015. Dr. Samathanam said that India's annual health expenditure is Rs.80,000 crores whereas both public and private sector spending on research is Rs.1,150 crores (1.4%). Medical research needs to be focused on therapeutic drugs/vaccines for tropical diseases which are normally neglected by MNCs on account of their limited profitability potential. Emphasis needs to be on the newly emerging frontier areas of research based on genetics, genome based drugs and vaccine development, molecular biology *etc*. He explained the agencies involved in funding the research and the various steps involved in the drug development process.

Dr. Samathanam then brought the attention of the participants to the different antibiotics available for therapeutic purposes and focussed on the emerging problem of NDM-1. He said that the threat of this group of bacteria destabilises the disease management strategy and makes it very complicated. He described that microbes developing resistance to antibiotics is a well-established truth. On the other hand, investment to develop new antibiotics is increasing with decreasing return of effective, safe, affordable and successful antibiotics. For a country like India to afford expensive, second line antibiotics is difficult. The unscrupulous use of first line antibiotics is generating incidences of resistance among the affected population especially for TB and other communicable diseases. He concluded his lecture by stating that there is an urgent need to review the use of antibiotics in the country, generation of field data along with infrastructure development to screen such antibiotic resistant populations as well as segregation/isolation/quarantine of such populations. He further emphasized that to achieve this we require to have academic/pharmaceutical/industry partnership in R&D through the various funding agencies that encourage public-private partnership R&D programmes in the Govt. of India.

**Dr. Sadhna Srivastava**, Scientist D, IPR Unit, Indian Council of Medical Research, New Delhi said that the antimicrobial resistance issue is not at the forefront in India. She outlined her presentation on the following areas: growing health concern especially in developing countries and least developed countries, no new antimicrobials coming in the market, very little interest by the pharma industries because it is not market driven, other options on how to promote development of new antimicrobials, some regulatory issues with special focus on counterfeiting medicines, national policy to combat the problem, possible suggestions to streamline the regulatory procedure and more importantly, the enforcement. She said that antimicrobial resistance is an alarming situation for communicable diseases. Further, no new antimicrobials are available in the market due to disinterest of the pharma companies. According to Professor Harrison, 1998 Nobel Laureate, antimicrobial resistance is not a new phenomenon but it is worrisome since this situation is accelerating and the tools for identifying how to combat antimicrobial resistance are not. This is not at the forefront for health policy makers. Due to MDR there is increase in morbidity, mortality and disability. The current scenario is still the same. The reason is that in countries like India we are entangled with problems with infectious diseases like tuberculosis, malaria, leishmaniasis, *etc*. therefore resistance due to antimicrobials is not the priority. In the last 5 years, 40% increase in cephalosporin sale and 60% increase in usage of antibiotics was reported by the Global Antibiotic Resistance Partnership (GARP) [7]. There are two extreme conditions. First, in India for the 4 lacs pneumococcal infections in children they are not able to get antibiotics. Secondly, there is irrational use of antibiotics in conditions like cold, cough and dysentery where it is of no use.

Counterfeiting in antimicrobials has added new dimensions towards resistance. The issue has magnified since there is no clear definition of counterfeiting. Further, it has generally covered sub-standard, spurious, misbranded fake drugs and generics under the same bracket which leads to regulatory and IP issues. She said that inexperienced pharmacists are responsible for the irrational use of antibiotics. Increasing drug pressure is the only reason for evolution of resistance and removal of drug pressure does not always lead to decrease in resistance. It is not only a medical problem but a cumulative problem, including social, cultural, economical, trade and global issues. Further, transmissible resistance (on plasmids) is especially problematic (classical example is NDM1 gene). Basically, bacteria borne resistance is more dominant and hospital and community acquired resistance is becoming more common. This is due to spurious/poor quality antimicrobials. Drug resistance is growing in the South East Asian Region with respect to malarial infection. About 95% of the population are at moderate to high risk in India. About 75% cases and over 50% deaths in the SEARO region have been identified from India. There is an alarming increase in chloroquine resistant *P. falciparum*. Sulfadoxin pyrimethamine resistance is on the rise. MDR *P. falciparum* has been reported. Deteriorating epidemiological indices is associated with drug resistance. Thus the whole scenario highlights the need for new antibiotics for malaria. She further added that the current global R&D is inadequate with respect to quantity and quality. The need for antibiotics will thus continue to be high and will increase because an ageing population needs new antimicrobials, increased global infection rates, increasing numbers of immuno-compromised patients and increasing bacterial resistance. Therefore, more R&D is required. The concerns of R&D for new antimicrobials are that most of the research is done in the US and Western Europe. Basic research is generally on the wane as funding is coming down, there are few new drugs, new drug pipelines are near-dry and companies find it uneconomical to bring out new antibiotics. Anti-microbials are not preferred by pharma companies because drugs for life-style diseases *viz*., heart disease, cholesterol-lowering agents, anti-inflammatories, diabetes mellitus or Alzheimer’s disease are taken for prolonged periods. Long term returns on anti-infectives has poor average revenue of antimicrobial amounting to less than $500 million. (less than the $1.0 billion needed by a company). Many older agents are still sold quite widely, as generics, giving reduced profits for the innovator companies. Patent rights for several antiinfective drugs have either expired or will soon, for instance co-amoxiclav (2001), ciprofloxacin (2004), clarithromycin, azithromycin and ceftriaxone (2005) and levofloxacin (2007).

She further explained the regulatory strategies in India by highlighting the fact that there is little or no regulation of antibiotics; particularly at pharmacies, susceptibility testing and drug resistance are difficult in rural settings and there is a need for national regulations which have just been framed by the Directorate General of Health Services, Ministry of Health and Family Welfare, Govt. of India [8]. She highlighted on the new policies for containment of antimicrobial resistance in India and stated that there are definite policies/guidelines for appropriate use of antimicrobials at national level in specific national health programs beings run in the country (*e.g*., RNTCP, national AIDS control program *etc*.). For other diseases with public health importance like enteric fever, diarrhoeal diseases, respiratory infections *etc.*, individual hospitals are following their own policies and hospital infection control guidelines. To monitor antimicrobial resistance, it is necessary to have: regulations for the use and misuse of antibiotics in the country, creation of a national surveillance system for antibiotic resistance, mechanisms for monitoring prescription and regulatory provision for monitoring the use of antibiotics in human, veterinary and industrial sectors and identification of specific intervention measures for rational use of antibiotics. She said that regulation is thus a grey area because there are no publications. Only 20% of WHO member states have some regulation on antimicrobials and these member states belong to the developed countries, and 30% have no drug regulation primarily represented by the developing and poor countries. Further there is no clear cut information for the counterfeiting whether it is intellectual property linked or public health linked or a TRIPS-plus measure.

The definition of counterfeit given by the WHO is very confusing and misleading because it is difficult to distinguish between spurious, substandard, misbranded and counterfeit. Counterfeit drugs have been defined as those which are deliberately or fraudently mislabelled with respect to identity/source or both. It applies both to brand and generic products; with correct or wrong ingredients, with or without sufficient active ingredients and with fake packaging including mislabelling. A substandard drug is a genuine drug but does not meet the quality specifications set for them. She emphasized that all the pharmaceutical products have to comply with quality standards and specifications before marketing. It should have adequate shelf-life required by the territory of use (*e.g.* high temperature in tropical countries) and should be reviewed, assessed and approved by National Regulatory Authorities. Dr. Srivastava concluded her talk by emphasizing that there is a need for streamlining regulatory procedures which can help in solving the problem of antimicrobial resistance. National policy has been initiated in India by Directorate General of Health Services and Ministry of Health and Family Welfare in 2011. Further, enforcement is a must for handling the menace and global support is required for this. She assured that the Indian Council of Medical Research, New Delhi has taken the initiative to prepare guidelines on issues related to antimicrobial and clinical trial registration and hopefully in 2-3 years some positive outcomes will be seen.

**Dr. Mufti Suhail Sayeed**, Vice President R&D and Head, Clinical Operations at the Venus Medical Research Centre, Panchkula, Haryana in his presentation explained that strategies and tools are required for the management of antimicrobial resistance rather than new drugs. This can be attained with the help of a translational approach. Dr. Sayeed said that there are 21 new antibacterial compounds in the pipeline from developing companies. Dalbovencin, which is in phase 3 of development, is considered to be one of the best drugs with a half-life of 10 days given once in a week. Dr. Sayeed explained that organisms vary in their antibiotic resistance pattern with respect to the environment and region. Antibiotic combinations have been a success for many years to address antibiotic resistance (AR) but still many antibiotic combinations need to be studied. Hence an adjuvant approach can be used to tackle the resistance problem. Antibiotic adjuvants are defined as antibiotic combinations with other strategies that enhance antimicrobial activity against the pathogen. These are molecules that block antibiotic resistance or antibiotic and non-antibiotic combinations. As per the research conducted in Venus Medical Research Centre, a novel molecule [VRP 1034 - Phytochemical] was discovered as an antibiotic adjuvant. This compound was reported to have the potential to inhibit efflux pump, stop biofilm formation, act as a catalyst and provide synergy to antibiotic combinations. It also makes the drug effective against ESBL producing strains, prevents mutagenesis by preventing conjugation, prevents resistance development and decreases infection induced hepatotoxicity with its antioxidant effect.

Dr. Sayeed said that regulatory measures have to evolve for guidelines to make adjuvants work. They are also reported to work better than –penams. He said that with the help of adjuvants, much of the effectiveness of antibiotics would be rejuvenated. He illustrated that novel combinations with VRP 1034 (Renisil) were found to increase the efficacy of antibiotics. As per the experiments conducted at Venus Research Centre, Renisil caters to all ESBL types *viz*., TEM, SHV, CTX-M and metallo-betalactamase, effectively. Expression of outer membrane protein was found to decrease with VRP 1034. The efficacy of Renisil was found to be at par with meropenem in *E. coli* and *K. pneumoniae* and superior with meropenem in *Pseudomonas aeruginosa*. The expression of Mex AB protein (OMP) in *P. aeruginosa* was much diminished with VRP 1034 adjuvant with antibiotic combination drug. It also found to break bacterial biofilm, prevent conjugational transfer of F-plasmid, alter cell membrane porosity and was considered safe as per the clinical studies conducted. Dr. Sayeed further stated that national and international clinical trials are still in progress. It is also known to reduce hospitalization time from 14 days to an average of 7 days and the pharmacokinetics of individual drugs are unaltered after adding VRP 1034. Improved safety and tolerability has been established by pre-clinical, Phase-III and Phase-IV studies. It further has the potential to replace Piperacillin, Tazobactum and save Meropenem as a reserve drug which is also manufactured in EU GMP approved facility.

## Major achievements

The conference on antimicrobial resistance grabbed the attention of academicians, clinicians, microbiologists, scientists, biotechnologists, agriculturalists, environmentalist, R&D personnel and industrialists to share a common platform to combat the menace. The event helped the understanding of the issues related to emergence and spread of antimicrobial resistance in a better way. National level data pertaining to the recent surveillance studies of various microorganisms was compared and analyzed. Specific intervention measures to harness the problem were discussed. It helped in uniting the network of people working on antimicrobial resistance to monitor the antibiotic susceptibility pattern among the clinical and environmental isolates. The event facilitated in identifying latest technologies that could help in understanding the mechanism of drug resistance and drug discovery.

## Proposed future action plan

The National Conference on Antimicrobial Resistance organized consecutively within a gap of three years gave us a clear picture on the present status and progress attained in this field at national level. As we are in pursuit of tackling this menace with those who work on the issue, we would like to further initiate network projects to generate surveillance data which will support in developing appropriate guidelines. As we have identified the key personnel and centres involved in the research on antimicrobial resistance, we expect the national bodies and funding agencies to support our projects on antimicrobial resistance so that we may effectively contribute in the future endeavours.

## Conclusion

**Prof. (Dr.) Rubina Lawrence**, Organizing Secretary and Convenor NCAR 2012 in her concluding remarks of the conference stated that from the deliberations made during these three days, four basic steps are needed in order to achieve the interrelated goals of antimicrobial stewardship and development—strategies that were discussed throughout the conference—and that have long been advocated to address the threat of AR. These are: limit the use of antimicrobials, discourage their misuse, reduce infection through disease prevention measures and create incentives for improved treatment and innovation. She further hoped that the initiative of this conference strengthens federal antimicrobial resistance surveillance, prevention, control and research efforts as well as enhances the collection of critical information on the use and misuse of antimicrobial agents. The Hon’ble Vice Chancellor **Most Rev.** (**Prof.) Rajendra B. Lal** during his concluding remarks appreciated the efforts taken by the organizing committee to unite national leaders and scientists in this field to find a plausible solution.

## Competing interests

The authors declare that there are no competing interests.

## Declarations

Publication of this meeting report was funded by the conference organizer, the Sam Higginbottom Institute of Agriculture, Technology and Sciences.

This article has been published as part of *BMC Proceedings* Volume 7 Supplement 3, 2013: Antimicrobial Resistance: A Cause for Global Concern. The full contents of the supplement are available online at http://www.biomedcentral.com/bmcproc/supplements/7/S3.
